# Accelerating the Passage to Citizenship: Marriage and Naturalization in France

**DOI:** 10.3389/fsoc.2021.659372

**Published:** 2021-05-10

**Authors:** Haley McAvay, Roger Waldinger

**Affiliations:** ^1^ University of York, York, United Kingdom; ^2^ UCLA Department of Sociology, Los Angeles, CA, United States

**Keywords:** citizenship, family, naturalization, France, marriage

## Abstract

Naturalization systems often provide immigrant spouses of citizens with accelerated access to citizenship, but thus far, the impact of such fast-track procedures has yet to be examined by empirical analysis. Toward that end, we leverage a unique feature of French naturalization policy: a dual track system, one for standard naturalization and a second that makes naturalization a right for non-citizens married to citizens. We show that, overall, family-level factors exercise the greatest influence on naturalization decisions relative to individual and contextual factors; further, marriage to French citizens is the single most powerful factor, yielding effects on naturalization in both tracks. However, while marriage to a naturalized citizen promotes standard naturalization, marriage to a French native fosters citizenship *via* the marriage track. Women migrants who marry French natives are particularly likely to naturalize *via* marriage. Contributing to the study of naturalization by attending to the link between two institutions—naturalization and marriage—we show that the effects of an apparent bias toward the familial ties of citizens run up against state efforts to close off membership to outsiders.

## Introduction

Recent scholarship on citizenship acquisition has increasingly focused on the micro context in which the naturalization decision unfolds: the family. Seeking to understand the decline in naturalization rates in Germany, Street (2014) notes that as family members are interdependent, individuals are likely to weigh family-level implications when deciding whether to acquire a new citizenship. Focusing on the Netherlands, Peters et al. (2016) emphasized that naturalization takes place in the “context of linked lives” (p. 361), tying the decision-making calculus of any one individual to the interests of other family members. Studying young adults who arrived in the United States as migrant children, Soehl et al. (2018) proposed an “embedded model of naturalization choice.” Their analysis complements Street’s, showing that just one variable—whether or not parents naturalized before the respondent or in the same year—has the single most powerful impact on naturalization.

Thus, family-level decisions can anchor or signal commitment to the country of immigration, whether by providing more information about the benefits of citizenship or the mechanics of the process, or by transmitting norms or values about civic membership. Yet not considered by this research are the institutional factors that also influence family effects on naturalization, as naturalization is constructed in ways that heighten the relevance of family interdependencies. In such countries as the United States, Spain, Norway, the Netherlands, France, and Austria, spouses of citizens enjoy an accelerated track to naturalization, gaining eligibility in a reduced time frame. Providing the foreign-born spouses of citizens with facilitated passage to citizenship reflects citizens’ greater claiming capacity, as well as the overall bias animating family reunification, which values the pursuit of citizens’ happiness. By facilitating naturalization of the foreign-born spouse, states protect the family life of citizen spouses, who gain the assurance that they and their spouse can forever remain on home grounds (Bonjour and Block, 2016). By privileging marriage to citizens, states also reinforce marriage’s importance while signaling an intuitive understanding of the lessons that migration scholarship teaches—that having intimate ties to citizens fosters integration (Abrams, 2013).

However, these very same procedures weaken states’ ability to control migrants and migration. Citizenship is a scarce status, wanted by many, in part because of the migration and mobility privileges it confers. As long as they lack citizenship, immigrant residents remain subject to the coercive power of the state, which can extrude them, prevent them from re-entering if they leave, and, by requiring them to renew residence permits, subject them to a type of continuing scrutiny from which citizens escape. As residence permits (such as lawful permanent residence in the Unites States) allow for long-term presence but do not provide the security gained *via* citizenship, the possibility of hastening the passage to citizenship by marrying a citizen can be the decisive influence on the marital decision, as some research suggests (Masure, 2014).

Naturalization also expands the pool of migrants who would not otherwise be eligible for entry, such as the parents of a naturalized spouse or the naturalized spouse’s minor children from a previous marriage. Furthermore, naturalization can accelerate family reunification: in some countries, such as the United States, access to residency rights for the spouses, parents, and minor children of citizens is a matter of processing delays, as opposed to the years postponing the arrival of denizens’ wives or husbands (Abrams, 2006). While acquisition of citizenship permanently protects naturalized immigrants from the threat of deportation, it similarly leaves them permanently free to benefit from the near-universal liberalization of divorce, separating from the citizen spouse and initiating a new, binational marriage, in turn triggering additional migration (Cole, 2014).

Consequently, procedures that facilitate the naturalization of citizens’ spouses weaken citizenship’s role as an institution of social closure. Moreover, as implementing those procedures activates a tension between two competing state goals—responding to, and validating, the preferences of citizens (who are also voters) vs. retaining tighter control over new entries—naturalization policy on the books and in practice may diverge. Heightening the possibility that the control imperative may take priority is awareness that tightened policies have left marriage as one of the few means of legal entry, and hence the growing perception that marriage comprises the weak link in the chain of migration control (Kringelbach, 2013). Not only is marriage inherently difficult to regulate, but the challenge is heightened by virtue of the fact that its control affects not only immigrants but citizens as well. Furthermore, as globalization spurs binational marriage, both *via* immigration *and via* the increasingly common, foreign experiences of citizens, many binational marriages occur abroad, thereby escaping home state supervision altogether.

Thus, “state agencies seeking to control and limit migration have marriage migration in their sights (Williams, 2012: 35).” Binational marriages increasingly fall under the suspicion that citizens and foreigners are using marriage instrumentally for immigration purposes (Beck-Gernsheim, 2007), leading migration control agencies to cast a dubious look at binational marriages. US Citizenship and Immigration Services, for example, reportedly views 20 to 33 percent of marriages between US citizens and immigrants as fraudulent (Brettell, 2017: 86). Consequently, requiring marriage migrants to demonstrate the bona fide nature of their relationship has become a pervasive aspect of migration control. While the legitimacy of binational marriages is often scrutinized at the moment of migration, later efforts to secure citizenship may reactivate those doubts, as indicated by a study of naturalization practices under the Trump administration, which found that adjudicators asked more questions about applicants’ marriage and demanded “more proof in the form of joint tax returns, bank statements, insurance and bills (Capps and Echeverria-Estrada, 2020:16).” Since officials have ample scope for discretion, the control imperative might also affect the criteria used to determine a marriage’s bona fides. Officials may view only certain types of applicants—by virtue of sex or national origin—or types of citizen spouses—by virtue of place of birth or parentage–as appropriate for benefiting from fast-track procedures. Similarly, relationships that depart from the standard pattern—for example, those involving a significant age difference between spouses—might induce additional scrutiny. Moreover, the strategic value of marriage for the purposes of migration can indeed generate marriages that might suffer from close examination, as suggested by the research of Boulahbel-Villac (1995), who profiled the pattern of younger, urban-origin, and better educated Algerian women marrying older, less educated, Algerian-born spouses residing in France. Thus, just as fear that close examination of one’s personal record might reveal problems better left hidden deters potential citizens from applying for naturalization (Gilbertson and Singer, 2003), so might concern over possibly problematic aspects of a marriage lead persons technically eligible for the accelerated track to opt for standard naturalization instead.

In this light, prior research on the familial embeddedness of naturalization may have overemphasized the importance of micro-level motivations at the expense of the match between two institutions—marriage and naturalization. Both marriage and naturalization bear a certain similarity, in that each entails a relationship to the state. A feature of many naturalization systems, the bias in favor of applicants with citizen spouses adds further incentives to acquire a new citizenship. However, whether qualifying applicants take advantage of fast-track procedures is an altogether separate question. Doing so necessarily puts the bona fides of marriages, as well as the documentation testifying both to the nature of the relationship and the identities of the partners, under closer inspection. Moreover, that heightened scrutiny occurs in a particular context: an immigration system biased toward exclusion and against noncitizens; a “securitization of immigration law and modernization of documentation systems increase(ing) the fear that documents are fraudulent (Mitchell and Coutin, 2019: 888)”; and a threshold event—namely, naturalization—making settlement permanent and foreclosing the possibility of expulsion.

This study represents the first empirical attempt to understand the impact of the institutional features of naturalization that, at least on paper, facilitate the route to citizenship for immigrants with citizen spouses. We do so by exploiting a distinctive feature of French naturalization policy. Although numerous countries facilitate the path to citizenship for the foreign-born spouses of citizens, France provides them with an altogether separate track. Naturalization by declaration, a procedure that, for simplicity, we will label “naturalization by marriage,” makes naturalization a right, thereby accelerating the process. We draw on the *Trajectories and Origins Survey* (TeO), a large, high-quality, representative survey of France’s foreign-born population, conducted in 2008–2009, which has the unique feature of distinguishing between these naturalization routes. Immigrants married to French citizens have much higher rates of naturalization (62 percent), than those married to foreigners (15 percent) and those without partners (39 percent). But surprisingly, most spouses of citizens do not naturalize *via* the marriage track, even if in principle they are all eligible to do so. Instead, when immigrant spouses of citizens naturalize, the great majority (77 percent) do so *via* the more time-consuming standard track, involving numerous interactions with authorities, during the course of which officials make an ad hoc assessment of the degree of assimilation.

Thus, going beyond previous research on the influence of family-level factors on naturalization decisions, this article seeks to illuminate the factors that propel naturalizing citizens onto one of the two different tracks, thereby gaining insight into the relationship between immigrants’ characteristics and the features of the system through which naturalization applications are processed. Using an event history analysis, we explore the determinants of naturalization by both tracks. We do so by considering the three levels on which prior scholarship has focused: the family level, focusing on factors related to the parentage of the spouse and the location of respondents’ parents and children; the individual level, focusing on characteristics such as age at migration, legal entry status, and education; and the contextual level, focusing on factors linked to migrants' countries of origins, which we retrieve from a variety of data sources and match to the TeO survey. Three main aims guide the analysis: 1) to explore whether naturalization determinants exert similar effects on both tracks, with a specific focus on marital status; 2) to assess the relative weight of individual-level, family-level, and contextual-level variables on naturalization over time; and 3) to assess whether marital status interacts with individual and contextual-level variables to put certain categories of migrants on differential pathways to citizenship.

## Background

### Naturalization in France

As a citizenship system, France, with its history as a *jus solis* system, low residency requirements, as-of-right citizenship for the spouses of French citizens, and acceptance of dual citizenship, resembles the liberal systems of settler states like the United States or Canada. Nonetheless, French naturalization rates are among the lowest in Europe.

Applications for naturalization begin at the prefecture, which sends accepted dossiers to the Interior Ministry for final determination. As broad national policies exercise influence at the Ministry whereas policing takes precedence at the prefecture, the Ministry decides whether an immigrant is naturalized, but the prefecture, in controlling the downstream paper flow, determines whether an immigrant can be a candidate (Spire, 2005).

Starting the process at the prefecture can be a deterrent (Spire, 2005): Applicants for naturalization would have previously visited the prefecture, often with unpleasant results, to obtain and renew residence permits (Mazouz, 2017). As everywhere, foreigners wanting citizenship need to put themselves under the microscope, which is why compiling a dossier of documents that fully establishes their identity and traces their trajectory from birth to the moment of application is an inherent part of the process. As the prefecture systematically requests reports on applicants from the police and security services, worries about a blemish on the record encourage postponement (Mazouz, 2017).

Waiting times are long and documentary requirements are exacting (Hajjat, 2013), with relevant information tightly rationed, leaving applicants uncertain about the information needed. As system attributes, the demanding nature of the requirements and the insistence on compliance simultaneously put the applicant to the “test of time”—indirectly testing the intensity of the applicant’s desire for naturalization—and signaling to the bureaucrat—*via* the ease or difficulty entailed in compiling the proper dossier—the degree of the applicant’s assimilation (Spire, 2005; Mazouz, 2017). These very same barriers weigh heavily on the low-skilled (Liebig and von Haaren, 2011).

Applicants must further satisfy requirements for cultural and social assimilation. Specified neither by law nor administrative rules, assimilation is subject to ad hoc interpretation. International migration entails the internationalization of families, yet French citizenship law mandates that France be at the center of the prospective citizen’s familial attachments. Consequently, agents tend to view applicants with families still in the homeland as ineligible, even if other criteria are fulfilled. As of the TeO survey, bureaucrats were charged with assessing linguistic assimilation yet lacked explicit criteria for determining needed competence levels. Consequently, attributes bearing no relationship to language ability often enter into a sphere where they do not belong, namely, consideration of an applicants’ degree of linguistic assimilation (Mazouz, 2017).

Instead of naturalization by decree, foreigners married to French citizens can follow a different track—naturalization by declaration, a procedure that makes naturalization a right. Weil (2002) described naturalization *via* marriage as largely open, although noting that 9 percent of the applications received by the ministry in 2003 were rejected. In reality, this track is encumbered. The extensive documentation required to naturalize by decree applies to naturalization by declaration, but in amplified form, involving documentation of the French-born partner’s nationality, two proofs of marriage, a criminal record summarizing all convictions handed down against the foreign spouse, an attestation of the continuity of marriage (with supporting documents), and a full birth certificate (Neyrand and M’Sili, 1995). Complying with even these basic requirements can prove problematic. Registry systems in developing countries remain incomplete: As of the early 2000s, according to UNICEF, more than a third of births worldwide went unregistered (Szreter and Breckenridge, 2012). As even the baseline requirements signal an underlying suspicion—as indicated by the demand for documents testifying to the continuity of the couple’s life together after marriage—“dossiers exclusively containing these required documents are rare. For the most part, they furnish complementary indications on the situation of the foreign spouse. One finds, for example, pay statements for the foreign spouse, work certificates, etc.” (Neyrand and M’Sili, 1995: 48). Despite the demand for proof of continuity of marriage, the prefect can undertake a “survey of morality,” inquiring not only into the bona fides of the marriage but also examining the degree of integration of the foreign spouse (as indexed by fluency in French) as well as the couple’s friendship patterns. Consequently, a significant measure of administrative discretion hovers over naturalizations occurring *via* the marriage track, which is why rather than escaping from the controls applied to naturalization by decree “in practice, it [the marriage track] sees itself submitted to the same criteria … as naturalization [by decree] (Masure, 2014: 203).”

The process has also become longer and more difficult over time. Up until 1993, a foreigner married to a French citizen could gain French citizenship by visiting the relevant authority (in most cases, the prefecture) and making a statement of intent to naturalize; presuming no objections, citizenship would then be granted after the following year. However, as marriages between foreigners and French citizens have grown increasingly suspect, tightening up on binational marriages became has increasingly been seen as an effective tool of strengthening migration control consequently, that waiting period was lengthened to 4 years, where it currently stands.

Last, for purposes of naturalization, the definition of marriage has deviated from the broader societal pattern. In France, long-term civil unions are increasingly common, recognized by law since 1999 as the legal equivalent of marriage; in 2008, only a few years after the institution of the *pacte civil de solidarité*, 265,404 marriages were concluded as compared to 137,766 civil unions, a gap that has narrowed significantly since. Whereas civil partnerships allow access to residency cards or family reunification (Sohler and Levy, 2009), only formal marriage permits spouses of French citizens to take advantage of the alternative, marriage track toward naturalization.

### Family-, Individual-, and Contextual Level Approaches

#### Family-Level Approaches

Research on familial influences emphasizes the ways in which the micro-level environment affects applicants’ motivations, in this respect building on a hypothesis earlier advanced by Yang (1994), who suggested that a greater commitment to life in the country of immigration may arise when both spouses are territorially present, thereby motivating the quest for citizenship. Similarly, Street (2014) hypothesized that for immigrant parents, the decision to naturalize would be heavily affected by the implications for their children. Thus, when the fate of immigrant parents and children was decoupled—with German citizenship attributed at birth to the German-born children of foreign-born parents, regardless of the latter’s citizenship status—naturalization among parents declined. Likewise, Soehl et al. (2018) demonstrated the interdependency of parents’ and children’s naturalization, yet also showed that the strength of that relationship diminishes with time, as evidenced by findings that influences from the parental household subside as children age and move out on their own.

In these studies, the migration of the core family network has been completed, with the crucial members in place in the society of destination. However, co-presence cannot always be presumed, as emigration often comprises a familial survival strategy, involving the short-term relocation of a single family member in order to consolidate income generating opportunities at home. Moreover, migration’s selectivity, leading younger persons to depart first, with dependents leaving later, or possibly never at all, ensures that international migration yields internationalized families.

These cross-border connections are likely to affect both migrants’ motivations to naturalize and perceptions by officials evaluating applications. Plans for return migration and continuing linkages with homeland kin, most importantly, spouses and children, may fortify homeland loyalties, leading eligible immigrants to select out of naturalization. Those very same ties may lead officials to perceive applicants with extensive transnational connections as unsuitable for citizenship, instead favoring those with strong family ties in France.

Politics also impinge on the relationship between migration and marriage, as marriage is a legal act, regulated by the state. Standard approaches conceptualize reduced social distance between immigrants and the mainstream as eventuating in inter-marriage. In turn, the diminished social distance denoted by marriage to a citizen could simultaneously signal a preference for citizenship and further generate the competencies needed to pursue that goal.

Since, as already noted, marriage can open access to both the territory and to citizenship, marriages between citizens and foreigners have become increasingly suspect. In France, the category of possibly dubious marriages has expanded from marriages fraudulently contracted for the purposes of residence or citizenship (“*marriages blancs*”) to marriages in which a foreigner fools a citizen partner into thinking that the marriage is motivated by love (“*marriages gris*”). Consequently, whether the officials reviewing an application perceive a marriage as suspect or genuine may depend on the characteristics and history of spouses and their relationship. Any number of traits—where the couples met, whether in France or abroad; where and when they married, whether before migration or after; the rootedness of the citizen spouse, whether naturalized or born in France; and whether the spouses are similar or different on such key attributes as age or education—may be enough to raise a red flag.

Consequently, family-level influences are likely to take varied form. Marriage to a citizen is likely to deepen the motivation to naturalize; however, fast-tracking naturalization also puts that marriage under the microscope, which is why characteristics of the relationship and of the spouse are likely to impinge on the route to citizenship. The broader set of family ties—to siblings and parents—comes into play as well, affecting decisions by both applicants and officials, with weaker connections to France possibly casting doubt on the marriage and hence reducing the likelihood that applicants will opt for the fast track.

#### Individual-Level Approaches

Family factors should therefore influence naturalization above and beyond the individual-level characteristics highlighted in prior literature. Neo-assimilation theory (Alba and Nee, 2003) contends that immigrants’ needs of survival compel adaptation, yielding skills that bring progress and exposure to “the mainstream.” In this view, the process leading to naturalization is one of linear change, with propensities growing as settlement generates resources. By contrast, human capital theory conceptualizes naturalization as an investment (DeVoretz and Irastorza, 2017), keying “citizenship ascension” to naturalization’s costs—language learning, fees, and validation of foreign degrees—and its benefits—the “citizenship premium.” Research points to the existence of a premium, though disentangling factors selecting for naturalization from those, net of selection effects, influencing naturalization’s rewards proves difficult. In France, naturalization has a powerful, positive effect on employment, especially among low-educated persons and women, who are particularly likely to be jobless (Fougère and Safi, 2009). The brevity of the French residency requirement, increasing the return to citizenship, as well as the goal of gaining employment to the large public sector, from which noncitizens are largely barred (Fougère and Safi, 2009) further add to the motivations to naturalize; however, the length, complications, and uncertainty of the naturalization process work in the opposite direction.

In seeking to control migration, states sift newcomers by legal status, which further structures options for naturalization. After the mid-1970s with the end to labor migration, legal entry mainly occurred *via* family reunification. Whereas workers or family members select the destination country as the target of migration, the destination country selects a small fraction of the world’s displaced for permanent residence; in choosing refugees or asylum-seekers, states subject them to close vetting, which also signals their deservingness.

Thus, prior research yields conflicting views regarding the channels linking individual characteristics to naturalization outcomes. While exposure should increase knowledge about acquisition, the longer the time spent without citizenship, the lower the pay-off. Likewise, naturalization may do most for the lowest skilled, a motivation possibly offset by difficulties encountered by poorly educated migrants navigating a complex. Less clear are implications for determinants of the naturalization track. As suggested earlier, characteristics at the relationship-, rather than individual-, level are likely to be the more important. Nonetheless, as allaying doubt is likely to ease suspicion, other sources of legitimacy—higher levels of education, entry with authorization, and refugee status—may favor naturalization *via* the faster track.

#### Contextual-Level Approaches: Country of Emigration Effects^1^

Naturalization involves a strategic decision, weighing the costs and benefits of the citizenship of the country of *emigration* against those of the country of *immigration*. Thus, immigrants from countries where political institutions function poorly should be more likely to naturalize, as the costs of citizenship loss are lower than for those from well-functioning democracies. Similarly, countries differ significantly in the degree to which their passports open doors internationally. The French passport has great utility as a travel document, providing visa-free access to 175 countries, in contrast to 56 for a Senegalese passport and only 47 for an Algerian passport.^2^

These considerations bear on the practical consequences of citizenship acquisition; other home country characteristics affect symbolic dimensions. Naturalization entails a transfer of national loyalties; prior socialization for membership in the home country people may impede that shift, as illustrated by the widespread belief among Latin American immigrants in the United States that the naturalization ceremony entails stomping on the home country flag (Jones-Correa, 1998). The historically fraught relationship between France and its former colonies, and Algeria in particular, may similarly lead the acquisition of French citizenship to be seen as an act of betrayal (Sayad, 1993; Beaud, 2018).

Beyond specific dyadic histories, a more general home country trait—the strength of national identity—can influence naturalization propensities. According to an analysis of the MGIS, “the more the national tie is perceived a strong affective tie, the more the change in nationality is a difficult decision to take and the fewer are those who take the step” (Tribalat, 1996, 168). Yet for immigrants from multi-ethnic states in sub-Saharan or central Africa where a strong national identity has not congealed, loyalty to the country left behind may be largely irrelevant.

Policies allowing dual citizenship can mitigate the loyalty problem, releasing immigrants to accept a second citizenship (Mazzolari, 2009). Since France accepts dual citizenship, sending country variation in dual citizenship policies are likely to matter, leading immigrants from countries that permit dual citizenship to be more likely to acquire receiving country citizenship than those that do not.

Overall, a disadvantaged context of origin should yield both material and symbolic advantages to naturalization, and hence motivate immigrants to acquire a new citizenship. Yet for precisely these reasons, background in a more disadvantaged context of origin might cast doubt on the legitimacy of efforts to pursue naturalization along the faster track.

## Data and Methods

Data come from the Trajectories and Origins (TeO) survey produced by INED/INSEE in 2008–2009 on a sample of over 21,000 respondents aged 18–60 years living in metropolitan France (Beauchemin et al., 2018). TeO overrepresents minority populations to ensure adequate-sized national origin subgroups.^3^ The survey includes detailed information on migratory trajectories, citizenship, and the type and timing of naturalization. Variables on respondents’ parents, spouses, and children shed further light on the family context.

We further enrich the TeO survey by matching respondents with information on their country of origin compiled from a variety of sources. This allows us to disentangle individual and family-level variables from country-level factors that influence the propensity to naturalize.

### Sample

Our analysis focuses on the immigrant population only who are either foreign or naturalized at the time of the survey.^4^ In France, immigrants are defined as foreign-born respondents without French citizenship at birth. There are 8,253 immigrants in TeO. As migrants only become eligible for naturalization after 5 years of residency, we exclude those who arrived in the 5 years prior to the survey date (*N* = 708, or 9% of all immigrants). To enable matching with country of origin characteristics, the sample is further restricted to migrants whose country of birth is reported in detail. This results in a sample of 6,411 migrants with 51 different national origins.^5^

### Modeling Strategy

There are two pathways to French citizenship^6^: acquisition through declaration and acquisition by decree. Naturalization through declaration is reserved for the spouses of French citizens. For clarity, we refer to this naturalization route as “naturalization through marriage.” Naturalization by decree is the more common track open to eligible foreigners. Out of the total 6,411 migrants in our sample, 35% naturalized by decree and 7% became French citizens by marriage (Table 1). The median time to naturalization was 11 years after arriving in France, but those who gained citizenship through marriage naturalized faster than those who naturalized by decree.

**TABLE 1:** Naturalization rates and timing.

	*N*	Weighted %
Naturalized by decree	2,373	35
Naturalized through marriage	461	7
Foreign	3,577	58
Total	6,411	100
—	Years from arrival	
Median time to citizenship	11	
*Via* decree	13	
*Via* marriage	7	

Due to the two-track naturalization system, our analysis employs a logistic discrete-time hazard model for multiple absorbing events. This estimation strategy is appropriate for event history analysis with two or more modes of failure, namely, naturalization by decree or naturalization by marriage^7^ (Rabe-Hesketh and Skrondal, 2008). We fit the model using a multinomial design into order to determine whether the independent variables shape the risk of naturalizing in different ways according to the track, with three possible outcomes: never naturalized, naturalized by decree, and naturalized through marriage. While the latter is only open to migrants with French spouses, all respondents are at risk of marrying over the period and then naturalizing by this route.^8^ Data were restructured into a person/year format, with each respondent having one observation for every year during which she is at risk of acquiring citizenship (*N* = 111,597). The observations begin the year respondents migrated and end once one of the naturalization events (or censoring) has occurred.

We built Model 1 including all individual, family, and contextual variables, selecting covariates measured prior to the naturalization event, or when the data allow, which vary over time. Table 2 provides summary statistics on all independent variables, described below. We interpret the model results as marginal effects of naturalizing in a given year by each track, holding all other values constant using Stata’s *margins* command. Due to repeated individual observations, the model is estimated using clustered standard errors at the individual level. We further replicated this model on a sample excluding European migrants (Model 2). Given free movement and residence within the European Union since 1992, Europeans may have a lower incentive to naturalize. We therefore ensure that our findings are not driven by this category of migrants.

**TABLE 2:** Summary statistics of covariates.

*Individual-level variables*		Mean
Generation	G1	0.57
	G1.25	0.13
	G1.5	0.14
	G1.75	0.17
Language ability	Spoke French during childhood (dummy)	0.31
Education	No education	0.27
	Primary school	0.09
	Middle school	0.10
	Vocational degree	0.17
	Professional bac	0.06
	General bac	0.08
	2-Year university degree	0.07
	Higher education	0.16
Employment status	Period(s) of unemployment since arrival (dummy)	0.22
Type of residency card	Asylum	0.06
	Student	0.10
	Worker	0.21
	Family reunification	0.36
	Exemption	0.08
	Other/unknown	0.19
Issuance of residency card	Card issued after arrival (dummy)	0.17
Migration trajectories	Migration to France before arrival (dummy)	0.18
	Migration abroad after arrival (dummy)	0.07
Demographics	Age	42.07
	Female (dummy)	0.51
***Family-level variables***		
Parental religion	Father or mother religious	0.93
Parents’ education	No education	0.66
	Primary/middle school	0.18
	Bac	0.05
	University degree	0.11
Children	Number of children born in France	1.68
	Number of children born abroad	0.40
Parents’ location	Not in France/unknown	0.57
	At least one parent arrived before R	0.30
	At least one parent arrived with or after R	0.13
Spousal characteristics	*Origin*	
	No partner	0.36
	Spouse is a French native with French native parents	0.15
	Spouse is French native with immigrant parent(s)	0.07
	Spouse is a naturalized French citizen	0.13
	Spouse is foreign-born	0.29
	More than 7 years age difference between spouses	0.20
	Premigration marriage outside of France	0.19
***Contextual-level variables***		
Country of origin	Polity score	0.37
	Citizenship loss in origin country (dummy)	0.39
	Former colony (dummy)	0.55
	Passport power	0.44
	Ethnic fractionalization	0.34

Next, we aimed to assess the relative influence of individual, family, and contextual variables on the likelihood of naturalizing by both tracks. We calculated predicted probabilities of naturalizing over time for migrants with “advantaged” vs. “disadvantaged” individual, family, and contextual characteristics. We define advantage and disadvantage empirically, based on the variables identified in Model 1 as favoring or impeding the likelihood of naturalization. Individual-level advantage is a migrant belonging to the G1.75 generation with the highest level of education. Individual-level disadvantage is a G1 migrant with no education. Family-level advantage refers to migrants with parents having the highest level of education, children and parents located in France, a spouse born in France to French-born parents (in the case of the marriage track), or a naturalized French spouse (in the case of the decree track). Disadvantage on family characteristics refers to migrants with parents lacking any education, children and parents not located in France, and having no spouse or a foreign spouse. We applied the same procedure to contextual characteristics to obtain predicted probabilities of naturalizing between migrants from advantaged contexts (strong polity, dual citizenship laws, strong passport power, not a former colony, and low ethnic fractionalization) and disadvantaged contexts (weak polity, no dual citizenship laws, low passport power, former colony, and high ethnic fractionalization).^9^ All other values were held constant.

Finally, we identified whether certain individual or contextual characteristics interact with the origin of the spouse in important ways for naturalization pathways, introducing interactions into the main model, described below.

### Family-Level Variables

#### Origin of the Spouse

Using the year of marriage and details on the origin of the spouse, we constructed a categorical measure of marital status. This measurement varies over time during the period at risk, so that we can chronologically ascertain the relationship between marriage and naturalization. About 70% of migrants in the sample are married, most of whom have an immigrant spouse, either naturalized (13%) or foreign (29%). 15% are married to French natives and 7% are married to French-born, second-generation immigrants. In the models, we group the “no spouse” and “foreign spouse” categories together.

#### Location and Timing of Marriage

For married respondents, we distinguished between migrants based on the location and timing of the marriage with a dummy variable: 1 for migrants married outside of France prior to migration and 0 otherwise.

#### Age Difference Between Spouses

We used a dummy capturing age differences between spouses, coded 1 if the spouses have a more than 7 years age difference and 0 otherwise.

#### Children

We identified whether respondents’ children were born in France or abroad. These variables are time-varying based on the year of birth, indicating the cumulative number of children born in France or abroad during the time at risk. On average, the sample shows more children born in France than abroad.

#### Parental Characteristics

TeO includes information on whether respondents’ parents have migrated to France and, if so, the time of their migration. We distinguished among parent(s) arriving before the migrant (30%); parent(s) arriving with or after the migrant (13%); and parent(s) not living in France at the end of the period at risk or whose place of residence is unknown (57%). We also included the educational level of respondents’ parents. As shown in Table 2, about two-thirds of respondents have parents with no education. Finally, we controlled for a dummy indicating whether either the mother or the father was religious.^10^

### Individual-Level Variables

#### Immigrant Generation

We constructed a 4-level immigrant generation variable based on age at migration. The G1 generation refers to migrants who arrived after the age of 17 years, G1.25 to those who arrived between the ages of 12 and 17 years, G1.5 to those who migrated between 6 and 11 years, and G1.75 generation to those who arrived as young children before 6 years of age.^11^ The large majority of the sample are G1 immigrants.

#### Language

A dummy indicates whether respondents spoke French during childhood (about one-third of the sample).

#### Education and Employment

We used an 8-level categorical measure of respondent’s education and the year of completed education. This measure varies over time during the period at risk. Levels of education are relatively low: Two-thirds did not obtain a high school diploma. A dummy indicates whether the respondent was ever unemployed during the time to naturalization (22% of the sample were at some point unemployed).

*Legal status upon arrival* is measured using information about the type of residency card and its date of acquisition. A categorical variable distinguishes among 6 statuses: refugees, students, workers, family reunification/French spouse, waiver, or other/unknown. A dummy indicates whether the first residency card was obtained after the year of migration, which would delay eligibility for citizenship. Most migrants arrived *via* family reunification (36%) or as workers (21%); 17% received a residency card late, that is, after their first year in France.

#### Migratory Trajectories

Two dummies capture migratory trajectories: *Migration before arrival* indicates a stay in France prior to arrival; *migration after arrival* indicates whether respondents had spent at least 1 year outside of France after arrival. 18% of the sample had been in France prior to immigration; 7% had lived in another country after immigrating.

#### Demographics

All models further control for gender, year, and year-squared.^12^ Given that we control for age at migration and that the clock starts upon arrival, the year variables can be interpreted as an effect of age on naturalization propensities.

### Contextual-Level Variables

Naturalization decisions are also influenced by the rights migrants stand to gain or lose by acquiring a new citizenship, a decision-making process which likely varies by country of origin. TeO reports the specific country of origin of migrants as well as their year of migration. This allows us to merge the TeO survey with additional data sources to retrieve contextual indicators relative to the country of origin at the time of migration to France.

#### Polity Score

We assigned each TeO respondent a polity score based on the relative strength of democracy in their country of origin. This variable comes from the Polity IV Project (Marshall and Jaggers, 2002) which ranks countries over time, allowing us to match respondents based on their country of origin and time of migration. The scale ranges from −10 (weak) to 10 (strong). We rescaled the variable from 0 to 1.

#### Ethnic Fractionalization

This variable comes from the Quality of Government Basic dataset (Dahlberg et al., 2021) and measures the strength of national cohesion in the country of origin. These data are also available over time, allowing us to match the information to TeO at the time of migration. Specifically, it measures the probability that two randomly selected individuals are not from the same ethnic group. Respondents tend to come from countries with somewhat weak polities on average (mean = 0.37) and moderate ethnic fractionalization (mean = 0.34).

#### Passport Power

Henley and Partners 2018 Passport Index ranks the visa-free travel freedoms provided by all countries, ranging from 1 (weak) to 91 (strong). We reversed the original scale so that higher values indicate greater passport power. As these data are current measurements and are not available over time, this measurement does not vary according to the time of migration. However, it is unlikely that countries’ passport power have changed substantially over time. We rescaled the variable from 0 to 1. The sample mean is 0.44, indicating moderate passport power in migrants’ countries of origin.

#### Citizenship Loss

We matched the TeO survey with the MACMIDE Global Expatriate Dual Citizenship Dataset (Vink et al., 2015) documenting dual citizenship policies for 200 countries since 1960. We created a dummy indicating whether the naturalization of a TeO respondent would have resulted in citizenship loss based on their country of origin. This variable was measured at the time of arrival in France. 39% of the sample were at risk of losing citizenship upon naturalizing in France.

#### Origins in Former Colonies

We recoded the country of origin variable reported in TeO into a dummy to indicate whether migrants emanate from a former French colony. This is true of about half of all respondents.

## Results

Table 3 shows naturalization rates according to individual, family, and contextual variables. Family characteristics are decisive to acquiring citizenship. Marriage is tightly intertwined with naturalization: As of the survey, only about one-third of unpartnered migrants possessed French citizenship.^13^ The origin and citizenship status of the respondent’s spouse produce the greatest variation in naturalization rates. 79 percent of respondents married to a naturalized French citizen are also naturalized, although most had obtained French citizenship by decree, not through marriage. While naturalization rates were lower among persons married to French-born children of French-born parents, naturalization *via* marriage was particularly common. By contrast, only 15 percent of respondents married to noncitizen, foreign-born persons had acquired French nationality.

**TABLE 3:** Naturalization rates by individual and contextual characteristics.

	% Not naturalized	% Naturalization by decree	% Naturalized via marriage
***Individual-level variables***			
*Generation*			
G1 migrated after 17 years	69	23	8
G1.25 migrated at 12–17 years	54	40	6
G1.5 migrated at 6–11 years	43	51	6
G1.75 migrated at 0–5 years	37	59	4
*R’s language during childhood*			
Foreign	62	31	7
French	50	43	7
*R’s education*			
No education	65	29	6
Primary schooling	68	27	5
Middle school	60	34	6
Vocational degree	51	42	7
Bac pro	47	46	7
Bac general	58	35	7
2-Year university degree	45	46	9
Higher education	51	39	10
*R experienced unemployment after arrival*			
No	59	34	7
Yes	53	39	8
*Residency card*			
Asylum	43	51	6
Student	52	36	12
Worker	73	23	4
Family reunion or married French citizen	59	32	9
Waiver	60	34	6
Other/missing	46	48	5
*Residency card issued after arrival*			
No	57	36	7
Yes	62	31	8
*Migration prior to arrival*			
No	56	38	7
Yes	68	22	9
*Migration after arrival*			
No	57	36	7
Yes	68	25	7
*Gender*			
Male	59	35	5
Female	57	35	9
***Family-level variables***			
*R’s parents’ education*			
No education	59	35	6
Primary/middle	54	38	8
Bac	57	34	9
University	59	32	9
*Number of children born in France*			
None	65	31	4
1, 2	56	35	9
3 or more	53	39	8
*Number of children born abroad*			
None	55	38	7
1, 2	69	23	8
3 or more	81	17	2
*R’s parents’ location*			
Unknown/not in France	67	25	8
At least one parent arrived before R	51	43	5
At least one parent arrived with or after R	35	57	8
*Origin of spouse*			
No partner	61	36	3
Spouse is French native with French parents	37	39	23
Spouse is French native with immigrant parent(s)	44	35	21
Spouse is naturalized French	21	73	6
Spouse is foreign-born	85	15	<1
Age difference between spouses			
Yes	57	33	10
No	58	35	6
Premigration marriage			
Yes	72	22	6
No	55	38	7
***Contextual-level variables***			
Citizenship loss			
No	57	36	7
Yes	59	33	8
Former colony			
No	65	28	7
Yes	52	41	7
Polity score			
<25th	49	45	6
25th–50th	45	44	10
>50th	70	24	7
Passport power			
<25th	52	41	7
25th–50th	52	40	8
>50th	64	29	7
Ethnic fractionalization			
<25th	66	27	7
25th–50th	55	39	6
>50th	54	39	8

Table shows row percentages.

The location of parents and children in France also matter to the likelihood of naturalizing. 65% of respondents whose parents migrated at the same time or after the respondent naturalized compared to 33% whose parents are not in France. Having at least one child born in France is associated with higher naturalization rates, while having children abroad is linked with lower chances of naturalizing.

Naturalization also varies greatly by individual characteristics, particularly age at arrival, education, and legal status. 63 percent of G1.75 and 57 percent of G1.5 respondents were naturalized (mainly *via* decree) as opposed to only 31 percent of those respondents who had arrived in France as adults. Respondents with the highest level of education were more likely to have gained citizenship than respondents who never went beyond primary school (49 percent vs. 32 percent), although higher levels of citizenship were actually obtained by persons with a 2 year university degree (55 percent). Status upon entry was a source of differences of comparable size, as 57 percent of persons admitted as asylum-seekers but only 27 percent of those who entered as workers had obtained citizenship as of the survey.

Contextual indicators are not as salient to naturalization patterns relative to family- and individual-level variables. The polity scale captured the widest differences: French citizenship had been obtained by only 30 percent of respondents originating in those states at or above the 50th percentile, as opposed to 54 percent among respondents from states at the 25th – 50th percentile and 51 percent among respondents from states at the 25th percentile or lower.

**FIGURE 1:**
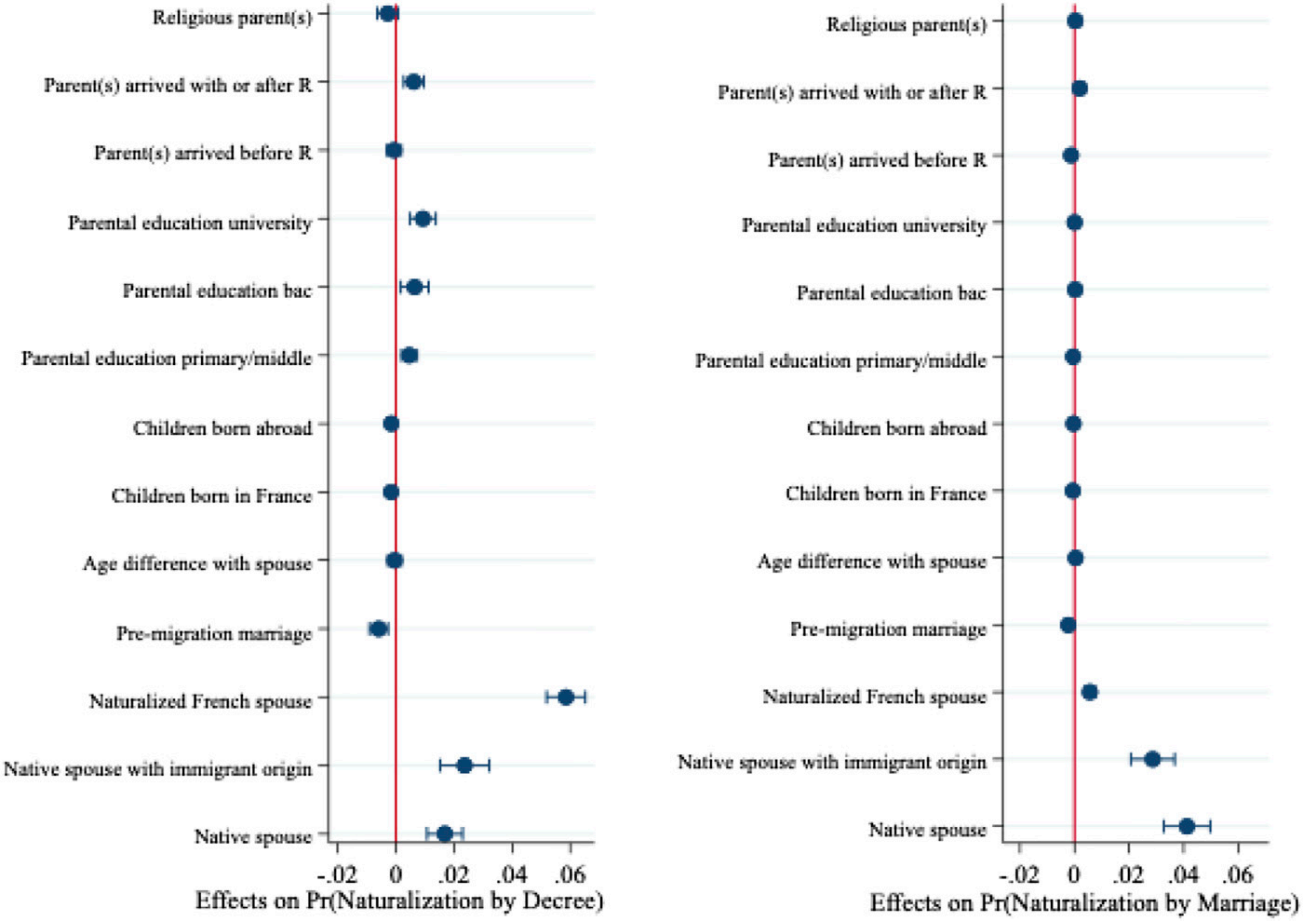
Marginal effects of family variables on naturalization from Model 1. Note: Reference categories for categorical covariates are as follows: origin of spouse (ref: no partner or foreign spouse); parental education (ref: no education); location of parents (ref: parent(s) not in France or unknown).

**FIGURE 2:**
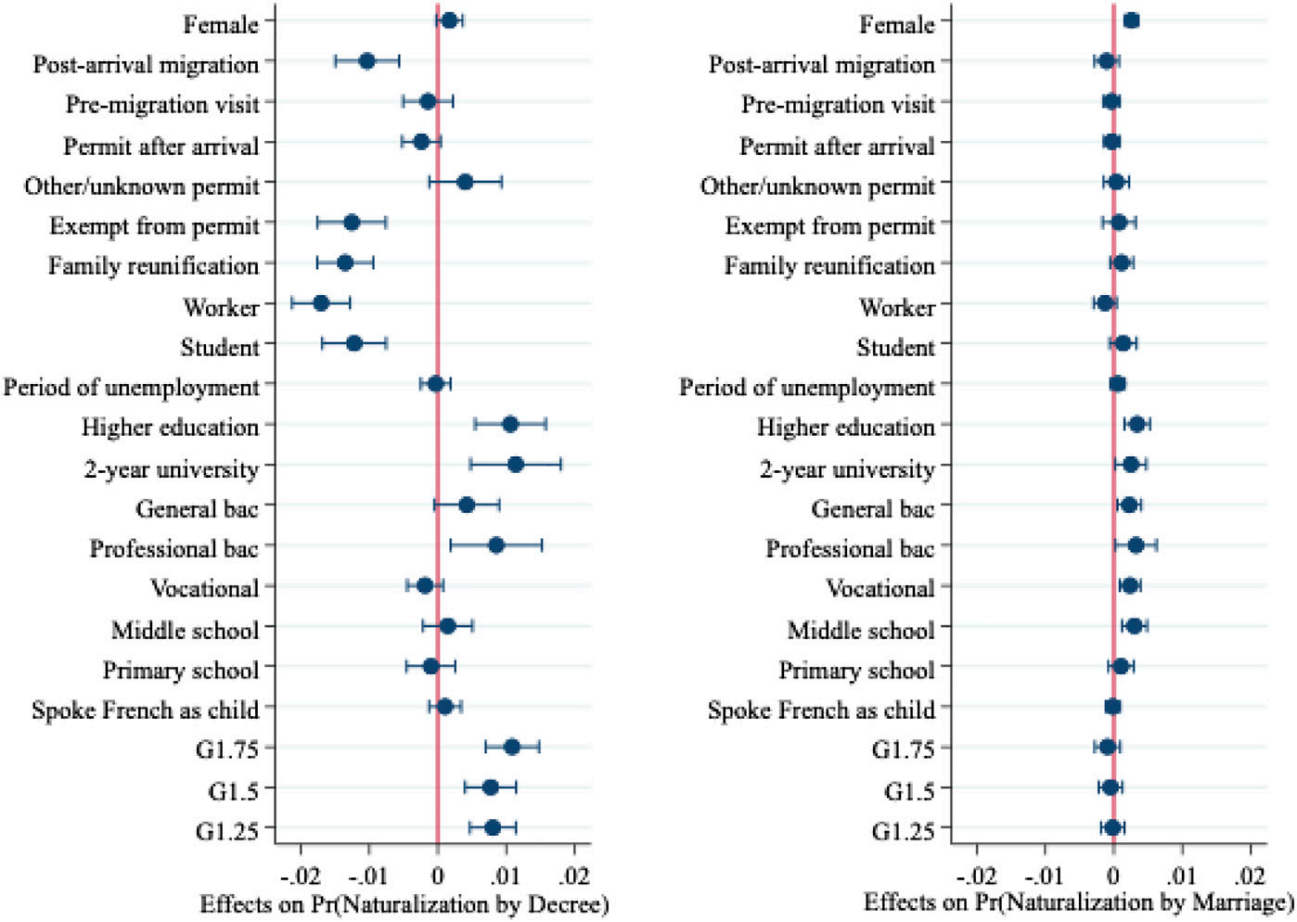
Marginal effects of individual variables on naturalization from Model 1. Note: Reference categories for categorical covariates are as follows: generation (ref: G1); education (ref: no education); residence permit (ref: asylum).

**FIGURE 3:**
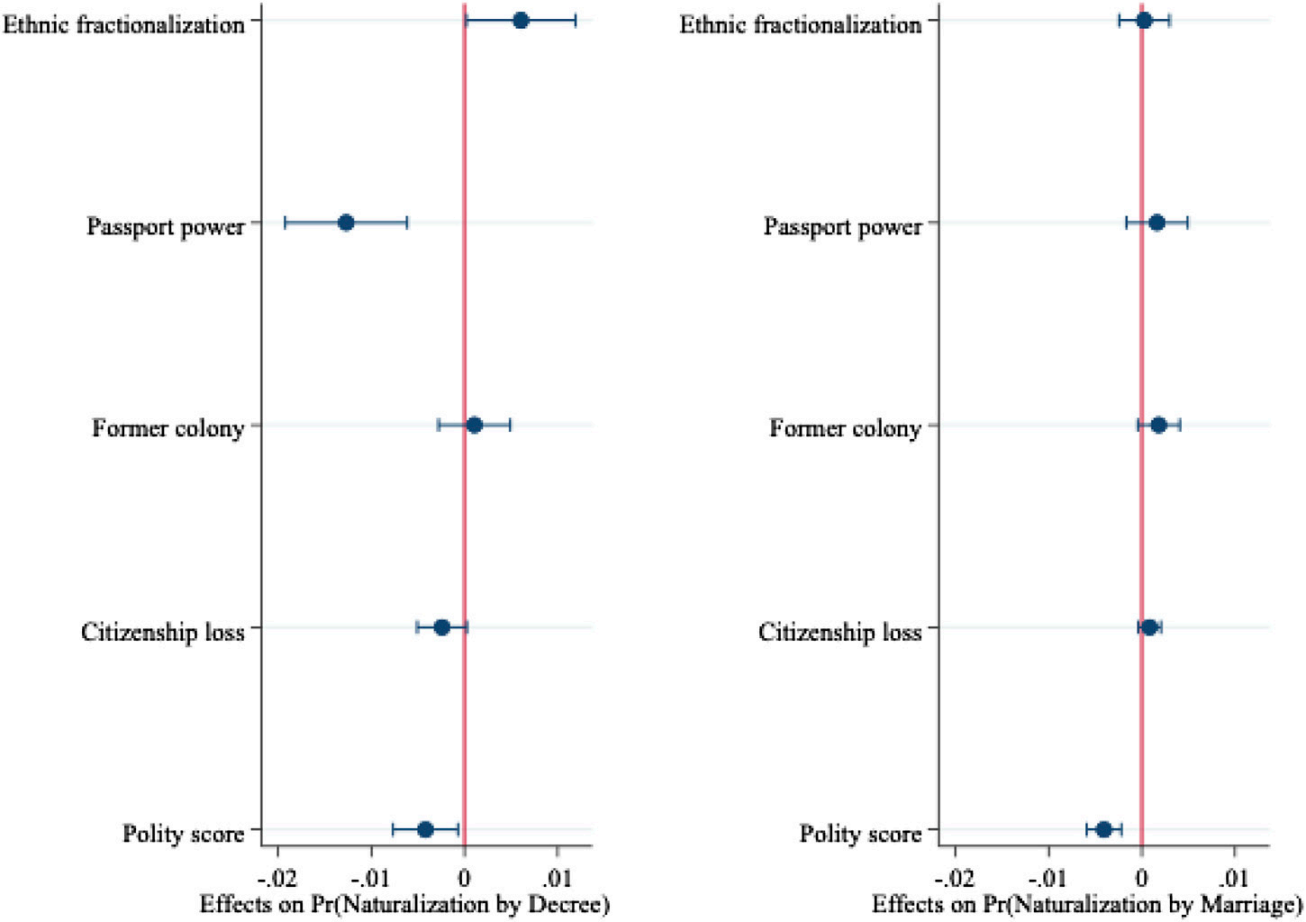
Marginal effects of contextual variables on naturalization from Model 1.

We ran a logistic discrete-time model with competing risks to test these individual, family, and contextual factors net of other factors. Results in Table A1 in the Appendix show the marginal effects of naturalizing in a given year by each track, separately for the full sample of migrants (Model 1) as well as for non-EU migrants (Model 2). To facilitate interpretation of the findings, we report the effects of individual, family, and contextual variables separately in Figures 1–3.

The results again highlight the importance of family characteristics (Figure 1), yet in contrasting ways according to the type of naturalization. Prior marriage to a French citizen promotes naturalization; this variable exerts the largest effect compared to all other covariates. However, the origin of the spouse plays out differently for naturalization by decree and naturalization by marriage. The probability of naturalizing by decree is highest for those with naturalized French spouses, whereas those married to natives (i.e., France-born spouses, born to France-born parents) are more likely to opt for the naturalization by marriage route. Marriages that occurred prior to migration outside of France negatively influence the likelihood of naturalizing by both tracks. A large age difference between the spouses does not, however, seem to matter. The location and education of parents also proves to be a significant predictor of naturalization. Migrants whose parents live abroad are less likely to naturalize than those with parents settled in France. Higher parental education also positively influences becoming French, although this variable only exercises influence on naturalization by decree.

Individual-level characteristics also have potent effects on naturalization but contribute more heavily to the naturalization by decree track (Figure 2). Very few individual variables matter to naturalization by marriage. Higher education accelerates access to French citizenship *via* both routes, although impacts are greater on naturalization by decree than by marriage. Immigrant generation matters to naturalization by decree, but is not decisive to naturalization *via* marriage net of other factors. Gender does not yield significant effects on naturalization by decree, yet women prove more likely to naturalize *via* marriage than men. Legal status upon arrival is also decisive for naturalization by decree, but neither legal status nor the timing of the first residency card affects citizenship through marriage.

Last, contextual variables play a minor role. Disadvantaged country of origin characteristics typically result in higher naturalization, but only the polity score–with those from more democratic polities less likely to obtain citizenship–yields any impact on naturalization by marriage.^14^

The majority of these findings are robust to the exclusion of Europeans (Model 2, Table A1). What’s more, the family and individual correlates of naturalization tend to be slightly stronger for non-European origins. Still, there are some notable differences with respect to Model 1. Immigrant generation is significantly related to naturalization by marriage for the non-European sample. Compared to migrants who arrived as adults (G1), migrants who arrived in childhood (G1.75 and G1.5) are less likely to opt for the marriage track. Non-European migrants who entered with a family reunification visa are also more likely to naturalize by marriage, suggesting that non-European migrants may more often draw on a pre-migration marriage with a French citizen to gain legal entry. Finally, not all contextual variables matter in the same way: The polity score loses significance among the non-European sample, whereas originating from a former French colony positively impacts naturalization *via* both routes.

**FIGURE 4:**
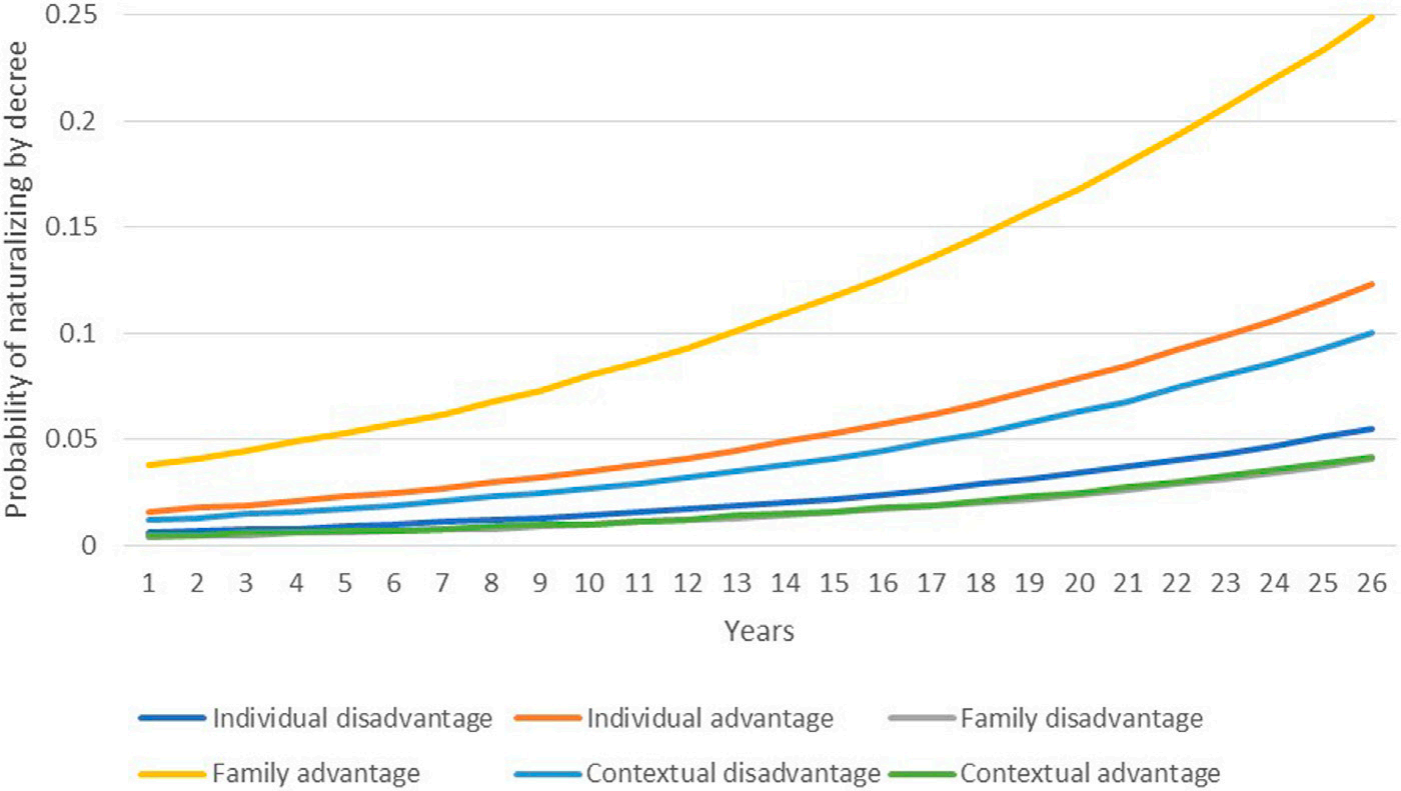
Predicted probabilities of naturalizing by decree according to changes in individual, family, and contextual variables.

**FIGURE 5:**
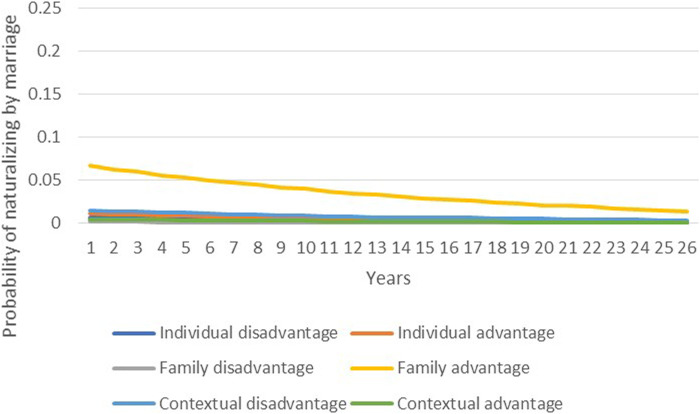
Predicted probabilities of naturalizing by marriage according to changes in individual, family, and contextual variables.

To test the relative weight of individual, family, and contextual variables, Figures 4,5 plot the predicted probabilities of naturalizing by decree and by marriage, respectively, based on disadvantaged and advantaged sets of characteristics. As Figure 4 shows, the probability of naturalization by decree is low in the early years following migration and then increases over time. Individual factors are powerful: After 26 years of residence in France, a 10 percentage point gap in the probability of naturalizing separates individuals with advantaged vs. disadvantaged characteristics. Yet, family advantage is an even more potent predictor, increasing the likelihood of obtaining citizenship by about 20 percentage points over 26 years. On the other hand, context plays a very small role, with minor differences between disadvantaged and advantaged contexts, and a contrasting pattern of impact: Migrants from disadvantaged contexts naturalize at higher rates than those from advantaged contexts.

As Figure 5 demonstrates, the likelihood of acquiring citizenship *via* marriage follows a different trend. Probabilities of naturalizing soon after migration are high and then decline as years in France increase, likely due to the fact that some migrants come to France with the intention to marry and naturalize quickly. In this naturalization procedure, family advantage again outweighs all other factors. While differences between individual and contextual variables are negligible, migrants with advantageous family characteristics have a 5 percentage point greater likelihood of naturalizing at the beginning of the period than migrants with disadvantaged family characteristics.

**FIGURE 6:**
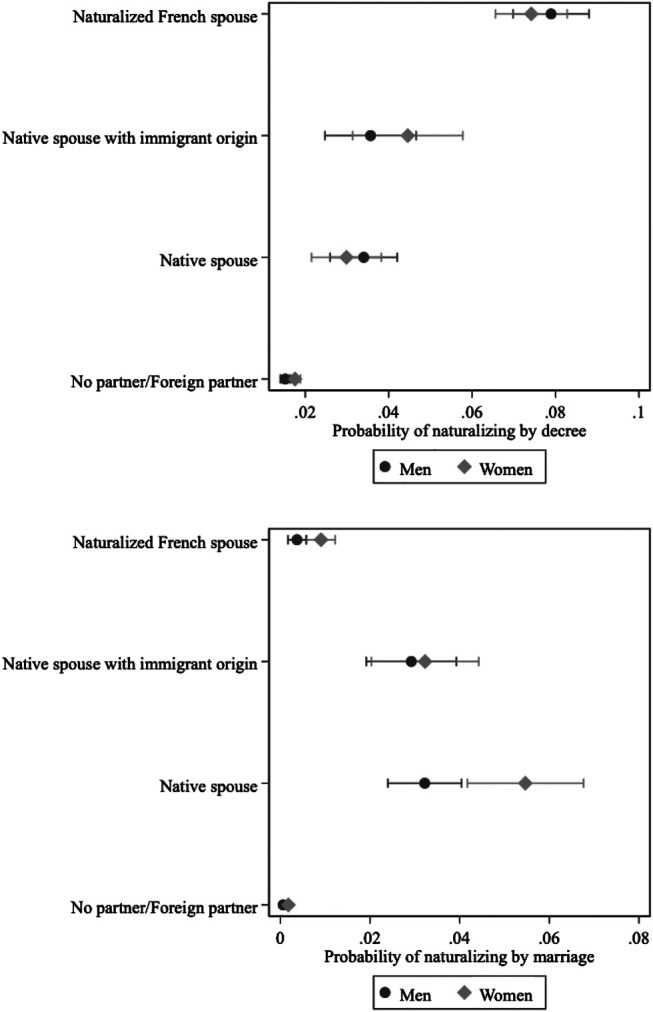
Interaction between gender and the origin of the spouse.

Finally, we aimed to assess whether the benefit of having a French spouse plays out similarly for men and women and across country of origin characteristics. Some groups may be more susceptible to administrative scrutiny during the naturalization process, particularly when naturalizing by marriage. We introduced two sets of interactions into the model: 1) between gender and spousal origin and 2) between migrant origin in a former colonial country and spousal origin. Figure 6 presents the gender and spousal origin interaction.^15^ Results suggest that women who are married to French native citizens with French parents appear to take the naturalization *via* marriage track to a greater degree than men. The interaction between former colonial country and spousal origin, however, did not produce significant results and is not shown here.

## Discussion and Conclusion

As Brubaker (1992) famously demonstrated, citizenship entails social closure. As an object of closure, citizenship is surrounded by obstacles that make its attainment elusive, even for resident non-foreigners who might enact and experience everyday citizenship. As an instrument of closure, citizenship generates inequalities between citizens and foreigners residing on the citizens’ territory. As a bias toward the familial ties of citizens characterizes both migration and naturalization policies, citizens’ own decisions to marry foreigners undermine states’ capacity to close off both territory and membership. Yet precisely because they represent the weak link in migration, the intimate ties between citizens and foreigners as institutionalized through marriage have increasingly become the focus of suspicion.

This study, drawing on the French *Trajectories and Origins Survey*, a rich, large-sample data set, has sought to build on earlier research demonstrating how family factors influence citizenship attainment. In doing so, we have also gone beyond that research, leveraging a distinctive trait of French naturalization policy to illuminate the factors allowing eligible immigrants to take advantage of fast-track procedures that facilitate naturalization for the spouses of citizens. To the best of our knowledge, this is the quantitative first empirical study to address this question.

Confirming prior scholarship, the study has shown that for the standard naturalization track—naturalization by decree—differences in citizenship take-up rates are strongly keyed to individual-level resources (Yang, 1994; Carrillo, 2015). Immigrants whose exposure to France started in childhood are more likely to naturalize than those who arrived later in life. Naturalization propensities rise with levels of education, although the main effects are felt at the high end of the spectrum, reflecting the stringencies of the process. Possessing a residency card at the moment of entry hastens passage toward citizenship; admission as a refugee or asylum-seeker is a still stronger accelerant. Migrant trajectories are also linked to naturalization decisions, with persons who remigrated after first arriving in France less likely to become citizens. While our analysis excludes by design persons who have permanently remigrated and cannot be observed, this finding indicates that migrants who stay in the sample are positively selected. By contrast, results for the standard track provide limited reinforcement for the importance of context. Country-of-origin effects on the standard track are keyed to disadvantage: Immigrants from countries that are less democratic and have passports that open fewer doors are more likely to naturalize, although these influences have very modest effects.

While these results largely confirm prior research, the study’s emphasis on the importance of family-level characteristics and, especially, its attention to the relevance of institutional factors yields new insight. Family-level traits prove even more powerful, in their effects on standard naturalization (naturalization by decree), than the individual-level characteristics to which previous scholarship has attended. By contrast, family-level influence almost entirely drives naturalization by marriage.

More importantly, we demonstrated that marriage to French citizens is the single most powerful factor, yielding effects on naturalization in both tracks. While couple formation is a social process, marriage entails a relationship to the state, which is why the very definition of marriage and its uses for the purposes of regulating immigration are instances of social closure. Like many other states of immigration, France grants the spouses of citizens greater access to citizenship, a pattern of exceptional treatment that has paradoxically made those marriages all the more suspect. The fact that only marriage allows potential citizens to access the marriage track at a time when other forms of nonmarital union are both increasingly common *and* state-sanctioned testifies to the social closure that surrounds citizenship and the distinctively political impediments to formal membership in the people.

Most of the married immigrant respondents were married to French citizens; nonetheless, usage of the marriage track proves uneven among those married to French citizens prior to naturalization. Most eligible persons forgo this route; the citizen spouse’s parentage proves to be the decisive factor in determining which option to choose. By contrast, a core assimilation variable such as generation bears no relationship to usage of the marriage track and education is barely influential with impacts only at the very highest end.

Here, we see the combination of the political and the social, reflecting the linkage between the status and identity dimensions of citizenship. Legally, naturalization by marriage is a right possessed by all immigrants with a citizen spouse; some immigrants do indeed exercise that right, but only if they have the right type of spouse, as indicated by the weak, almost negligible effects associated with individual and contextual factors. As persons choosing the marriage track have to comply with the extensive requirements needed to demonstrate the “truth” of their marriage, it is not surprising that the marriage option is far more likely to be selected by immigrants married to French citizens of French ancestry, as opposed to their counterparts with a naturalized citizen spouse, whose immediate foreign origin may be a source of suspicion. Likewise, our finding that women are more likely to use the marriage track than men suggests that the same shadow that makes the migration of foreign men married to citizens suspect—as well documented in the literature—extends to the naturalization sphere as well—which has not been previously shown.

In the end, naturalization is linked to states’ monopolization of the means of admissions, a process which includes their monopoly over the means of mobility, as Torpey (2018) has emphasized, but extends to their monopoly of what Walzer (1983) called “second admissions,” namely, naturalization. Controls at the first level discourage many would-be immigrants, but not quite as many as rich democracies like France would like, which is why leakage across the frontier always occurs. But errors or oversights at the first level can be corrected at the second level, as the techniques that suffice for entering the territory have no traction on naturalization, a sphere that is the province of the state alone. As foreigners’ entry into citizenship gives them a permanent place in the national landscape while also entailing easier first admissions for their relatives still living at home, the inherent connection between immigration and naturalization leads citizenship to be an increasingly elusive prize.

## Data Availability

Publicly available datasets were analyzed in this study. The data can be found here: http://quetelet.progedo.fr/donnees-francaises.

## Footnotes

1 The literature also draws attention to the relevance of country of immigration effects. In separate analysis, available upon request, we ran analyses including two such measures: one measuring support for the extreme right-wing party, Front Natonal, at the regional level, hypothesizing that naturalization probabilities will be lower where the party enjoys a high level of support; and a second measuring average educational achievement by national origin, hypothesizing that higher group-level resources will be associated with higher naturalization probabilities. As neither variable yields effects on naturalization by marriage nor affects other independent variables of interest, we dropped this discussion for reasons of concision.

2 Data drawn from the passport index https://www.passportindex.org [accessed August 21, 2017].

3 All descriptive analyses apply appropriate sampling weights.

4 While some second-generation immigrants born in France without French citizenship are also at risk of naturalizing, we exclude these respondents from our analysis as their citizenship acquisition is governed by a specific jus solis procedure.

5 TeO provides precise national origins for most groups. However, when sample sizes are small, certain origins are aggregated into larger categories (i.e. other Asia) and detailed country of origin is not provided; these respondents are excluded from the analysis. We included migrants from the following countries: Algeria, Morocco, Tunisia, Senegal, Mauritania, Gambia, Guinea-Bissau, Guinea, Mali, Burkina Faso, Niger, Chad, Cote d’Ivoire, Ghana, Togo, Benin, Nigeria, Cameroon, Central African Republic, Gabon, Congo (Brazzaville), Democratic Republic of Congo, Equatorial Guinea, Vietnam, Cambodia, Laos, Turkey, Portugal, Spain, Italy, Greece, Austria, Germany, Luxembourg, Denmark, Sweden, Finland, United Kingdom, Ireland, Belgium, the Netherlands, Bulgaria, Cyprus, Estonia, Hungary, Latvia, Lithuania, Malta, Poland, Czech Republic, Romania, Slovenia, and Slovakia.

6 Excluding cases of jus sanguinis, for people with French filiation.

7 We opt for logistic discrete time models over Cox proportional hazard models because we lack a fine-grained measure of the date of naturalization, only the year in which it occurred. Logistic discrete time models are also preferable when the data have many ties, i.e. many individuals are experiencing the event at the same time (Allison, 1982).

8 Because naturalization by marriage concerns a selected population, we ran two additional models: a model restricting the sample to respondents who are or were ever married, and a Heckman probit model where the selection equation predicts whether respondents are or were ever married. Findings and details on these models are presented in Table A2 in the Appendix. The main findings relating to the origin of the spouse are robust to these specifications.

9 We set the contextual characteristics at the minimum and maximum values; results also replicate when we use the 25th and 75th percentile values.

10 The available data only contain parental religiosity but do not report parental religion.

11 Some migrants who arrived in France as children may have naturalized before the age of 18 years, specifically if one of their parent(s) naturalized. To ensure that our results are not sensitive to these early naturalizations, we ran models excluding these respondents. 6% of the sample naturalized as minors (*N* = 418). Results do not change substantially when these respondents are dropped, with the exception that the effects of generation and parental education on naturalization by decree become insignificant.

12 We also ran the model controlling for dummies for each year. Results are robust to this specification.

13
Table 3 shows that a small percentage of unpartnered migrants naturalized by marriage. These respondents are predominately ex-spouses of French citizens. As we do not have the date of divorce/separation, we cannot chronologically order this event with respect to naturalization. However, given the small number of respondents concerned, it is unlikely that this substantially influences the findings.

14 We also estimated a model using clustered standard errors at the country of origin level. This did not alter the estimates of the individual and family-level variables. Some of the contextual variables lose significance. Disadvantaged country-of-origin characteristics are not significantly correlated to naturalization by degree. However, we still find a negative significant effect of the polity score on naturalization by marriage. We do not include this model for sake of concision, but the results are available upon request.

15 Model results including interactions are available upon request.

## Appendix

**TABLE A1:** Marginal Effects of Naturalizing by Both Tracks (Models 1 and 2)

	Model 1		Model 2	
	*Full sample*		*Excluding European nationals*	
	Naturalization by decree	Naturalization by marriage	Naturalization by decree	Naturalization by marriage
***Individual-level variables***				
Year	0.002***	−0.000***	0.003***	−0.000**
	(0.000)	(0.000)	(0.000)	(0.000)
Year squared	−0.000***	0.000	−0.000***	0.000*
	(0.000)	(0.000)	(0.000)	(0.000)
*Generation*/Ref: G1				
G1.25	0.008***	−0.000	0.011***	−0.001
	(0.002)	(0.001)	(0.003)	(0.001)
G1.5	0.008***	−0.000	0.008**	−0.002*
	(0.002)	(0.001)	(0.003)	(0.001)
G1.75	0.011***	−0.001	0.012***	−0.004***
	(0.002)	(0.001)	(0.003)	(0.001)
R spoke French during childhood	0.001	−0.000	−0.000	−0.001
	(0.001)	(0.001)	(0.002)	(0.001)
*R’s education*/Ref: No education				
Primary	−0.001	0.001	−0.003	-0.001
	(0.002)	(0.001)	(0.003)	(0.001)
Middle	0.001	0.003**	0.002	0.003**
	(0.002)	(0.001)	(0.003)	(0.001)
Vocational	−0.002	0.002**	−0.003†	0.002†
	(0.001)	(0.001)	(0.002)	(0.001)
Professional bac	0.009*	0.003*	0.012*	0.004†
	(0.003)	(0.002)	(0.005)	(0.002)
General bac	0.004†	0.002*	0.006†	0.003**
	(0.002)	(0.001)	(0.003)	(0.001)
2-Year university degree	0.011***	0.003*	0.015**	0.005**
	(0.003)	(0.001)	(0.005)	(0.002)
Higher education	0.010***	0.003***	0.017***	0.005***
	(0.003)	(0.001)	(0.004)	(0.001)
R experienced unemployment after arrival	-0.000	0.001	-0.000	0.001
	(0.001)	(0.001)	(0.002)	(0.001)
*Residency card on arrival*/Ref: Refugee				
Student	−0.012***	0.001	−0.017***	0.001
	(0.002)	(0.001)	(0.003)	(0.001)
Worker	-0.017***	-0.001	-0.021***	-0.001
	(0.002)	(0.001)	(0.003)	(0.001)
Family reunification	−0.014***	0.001	−0.017***	0.003**
	(0.002)	(0.001)	(0.003)	(0.001)
Exemption	−0.013***	0.001	−0.017***	0.002
	(0.003)	(0.001)	(0.004)	(0.002)
Other/unknown	0.004	0.000	0.004	0.001
	(0.003)	(0.001)	(0.004)	(0.001)
Residency card issued after first year of arrival	−0.002†	−0.000	−0.004*	0.000
	(0.001)	(0.001)	(0.002)	(0.001)
Migration before arrival	−0.001	−0.000	0.002	0.002*
	(0.002)	(0.001)	(0.003)	(0.001)
Migration after arrival	−0.010***	−0.001	−0.012**	−0.000
	(0.002)	(0.001)	(0.004)	(0.001)
Female	0.002†	0.003***	0.002	0.002**
	(0.001)	(0.000)	(0.001)	(0.001)
***Family-level variables***				
*Marital status*/Ref: No partner or foreign partner				
Spouse is French native with French parents	0.017***	0.041***	0.017***	0.041***
	(0.003)	(0.004)	(0.005)	(0.005)
Spouse is French native with immigrant parent(s)	0.024***	0.029***	0.027***	0.024***
	(0.004)	(0.004)	(0.006)	(0.004)
Spouse is naturalized French	0.059***	0.006***	0.063***	0.006***
	(0.003)	(0.001)	(0.004)	(0.001)
Married before migration	−0.006***	−0.002***	−0.008***	−0.002*
	(0.002)	(0.001)	(0.002)	(0.001)
More than 7 years of age difference between spouses	−0.000	0.000	−0.002	−0.000
	(0.001)	(0.001)	(0.002)	(0.001)
Cumulative number of children born in France	−0.002***	−0.001*	−0.002**	−0.000
	(0.000)	(0.000)	(0.001)	(0.000)
Cumulative number of children born abroad	−0.002*	−0.000	-0.001	-0.000
	(0.001)	(0.000)	(0.001)	(0.000)
*R’s parents’ education*/Ref: No education				
Primary or middle	0.005***	−0.000	0.008***	−0.001
	(0.001)	(0.001)	(0.002)	(0.001)
Bac	0.006**	0.000	0.008*	0.001
	(0.002)	(0.001)	(0.003)	(0.001)
University	0.009***	0.000	0.013***	-0.000
	(0.002)	(0.001)	(0.003)	(0.001)
*Parents’ location*/Ref: Parents’ not in France or unknown				
Parent(s) arrived in France before R	−0.000	−0.001*	−0.003	−0.002**
	(0.001)	(0.001)	(0.002)	(0.001)
Parent(s) arrived with or after R	0.006***	0.002†	0.006*	0.004*
	(0.002)	(0.001)	(0.003)	(0.002)
Mother or father religious	−0.003	0.000	−0.003	0.001
	(0.002)	(0.001)	(0.003)	(0.002)
***Contextual-level variables***				
Polity score	−0.004*	−0.004***	0.005	−0.002
	(0.002)	(0.001)	(0.004)	(0.001)
Citizenship loss	−0.002†	0.001	−0.004†	-0.000
	(0.001)	(0.001)	(0.002)	(0.001)
Former colony	0.001	0.002	0.007*	0.004*
	(0.002)	(0.001)	(0.003)	(0.002)
Passport power	−0.013***	0.002	−0.016†	0.001
	(0.003)	(0.002)	(0.009)	(0.004)
Ethnic fractionalization	0.006*	0.000	0.002	0.003
	(0.003)	(0.001)	(0.004)	(0.002)
Observations	100,194	100,194	60,966	60,966

Standard errors in parentheses.

****p* < 0.001, ***p* < 0.01, **p* < 0.05, ^†^ *p* < 0.10

**TABLE A2:** Robustness Tests of the Origin of the Spouse Effect

	Basic Model 1		Restricted to respondents who are or were ever married		Heckman probit models	
	Naturalized by decree	Naturalized by marriage	Naturalized by decree	Naturalized by marriage	Naturalized by decree	Naturalized by marriage
Ref: No partner/foreign partner						
Spouse is French native with French parents	0.792***	3.679***	0.780***	3.425***	0.324***	0.769**
	(0.102)	(0.166)	(0.104)	(0.168)	(0.041)	(0.289)
Spouse is French native with immigrant parent(s)	0.975***	3.312***	0.988***	3.070***	0.429***	0.694**
	(0.118)	(0.187)	(0.119)	(0.186)	(0.051)	(0.258)
Spouse is naturalized French	1.635***	1.839***	1.654***	1.613***	0.747***	0.311*
	(0.059)	(0.191)	(0.059)	(0.188)	(0.029)	(0.129)
*N*	100,194	100,194	82,348	82,348	100,194	100,194

Robust standard errors in parentheses.

****p* < 0.001, ***p* < 0.01, **p* < 0.05, ^†^ *p* < 0.10

Table shows coefficients. All models control for the same set of covariates included in Model 1. The selection equation of the Heckman probit models predicts whether the respondent is or was ever married and includes the following covariates: year, year squared, generation, language spoken during childhood, educational level, unemployment, gender, and parental education.
